# Nitrile Synthesis with Aldoxime Dehydratases: A Biocatalytic Platform with Applications in Asymmetric Synthesis, Bulk Chemicals, and Biorefineries

**DOI:** 10.3390/molecules26154466

**Published:** 2021-07-24

**Authors:** Pablo Domínguez de María

**Affiliations:** Sustainable Momentum, SL, Av. Ansite 3, 4–6, 35011 Las Palmas de Gran Canaria, Canary Islands, Spain; dominguez@sustainable-momentum.net; Tel.: +34-6-0956-5237

**Keywords:** biocatalysis, green chemistry, nitriles, aldoxime dehydratases, sustainability

## Abstract

Nitriles comprise a broad group of chemicals that are currently being industrially produced and used in fine chemicals and pharmaceuticals, as well as in bulk applications, polymer chemistry, solvents, etc. Aldoxime dehydratases catalyze the cyanide-free synthesis of nitriles starting from aldoximes under mild conditions, holding potential to become sustainable alternatives for industrial processes. Different aldoxime dehydratases accept a broad range of aldoximes with impressive high substrate loadings of up to >1 Kg L^−1^ and can efficiently catalyze the reaction in aqueous media as well as in non-aqueous systems, such as organic solvents and solvent-free (neat substrates). This paper provides an overview of the recent developments in this field with emphasis on strategies that may be of relevance for industry and sustainability. When possible, potential links to biorefineries and to the use of biogenic raw materials are discussed.

## 1. Aldoxime Dehydratases as Biocatalysts for the Cyanide-Free Synthesis of Nitriles

**Figure 1:**
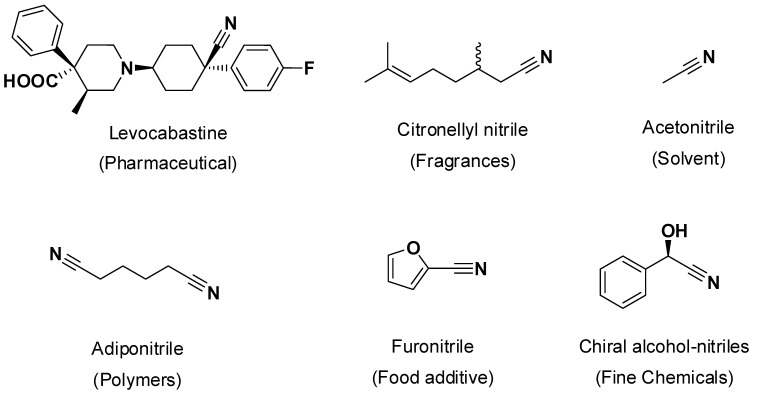
Selected examples of occurrence of nitriles with different uses as solvents, pharmaceuticals, food additives, or as polymer precursors [1,2,3].

Nitriles comprise an important group of chemicals that are widely spread in industry in a broad range of sectors, being used as products, solvents, polymers, commodities, or as starting materials for the production of other chemicals such as amines, amides, etc. (Figure 1). A nitrile group (–C≡N) displays a linear geometry with sp hybridization of the triple bond. Depending on the application, their annual production may range from kilo scale low-volume, high-value applied to pharmaceuticals and fine chemicals, to millions of tons worldwide for solvents and commodities [1,2,3]. Several synthetic routes have been proposed for the industrial production of nitriles, typically starting from petroleum-based alkenes (e.g., ethene, butadiene), and proceeding via hydrocyanation (using hazardous (hydrogen) cyanide), or via ammonoxidation (using ammonia and alkanes), or via amide dehydration [1,2,3]. These syntheses have been successfully established at a commercial level and largely optimized over decades.

The quest of sustainable industrial chemical strategies that may serve as alternatives to current processes is nowadays a matter of intense research. Some approaches focus on using renewable resources as starting materials, such as lignocellulose, CO_2_, wastes, etc., as well as (bio)catalytic alternatives that may lead to more environmentally friendly production in terms of higher atom economy, less waste production, and energy savings [4,5]. With respect to nitriles, developing versatile cyanide-free routes that could be (preferably) performed using biogenic resources and under mild reaction conditions are of particular interest. Hence, biocatalysis may become an important alternative, provided that optimized enzymatic routes are set with high substrate loadings and productivities aligned with industrial interests [5,6,7,8,9,10].

**Scheme 1:**
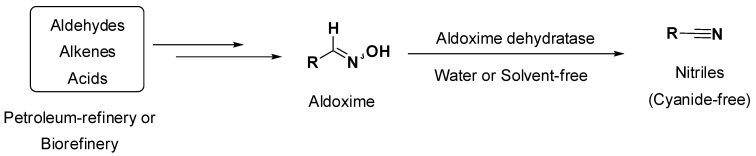
Conceptual approach using aldoxime dehydratases to afford nitriles under mild reaction conditions.

Over the last years, a biocatalytic platform for the synthesis of nitriles has emerged as a promising alternative in terms of sustainability (use of renewable resources, cyanide-free, mild conditions) and industrial interest (high productivity and substrate loading). Aldoxime dehydratases catalyze the dehydration of oximes in several reaction media, affording nitriles at mild temperatures and ambient pressure. The used substrate aldoximes can be in situ (and straightforwardly) formed via condensation of hydroxylamine with aldehydes, which can be derived either from classic petroleum refineries (e.g., hydroformylation) [1,2,3] or from renewable resources such as alcohols or carboxylic acids, among other options [4] (Scheme 1).

The first aldoxime dehydratases were characterized two decades ago by the Asano group when working with soil samples and isolating microorganisms able to grow with aldoximes as the carbon source [11,12,13]. Aldoxime dehydratases belong to the enzyme class of lyases and do not need a cofactor (although they carry a heme B group within the active site). Over the last few years, the Asano and the Gröger groups have jointly established a very versatile and industrially sound platform using aldoxime dehydratases for the synthesis of nitriles, identifying a number of active biocatalysts [14] and a broad substrate spectrum ranging from chiral nitriles to bulk chemical applications using several reaction media such as aqueous, organic solvents, or even solvent-free systems [15,16,17]. In the following sections, selected recent examples of synthetic applications of aldoxime dehydratases will be showcased. Emphasis will be put on the practical use of the enzymes together with sustainability aspects and potential links to biorefineries. Overall, the topic represents a fascinating example on how biocatalysis can align industrially sound applications with promising sustainability metrics [9].

## 2. Applications of Aldoxime Dehydratases in the (Asymmetric) Synthesis of Fine Chemicals, Fragrances, and Pharmaceuticals

When establishing a new biocatalytic platform, the first intended applications are often related to the asymmetric synthesis of optically active chemicals, making use of the exquisite enantioselectivity that most of the enzymes display [5]. As enzymes catalyze reactions in an active site (a cavity within the protein scaffold, which is, by definition, a multi-chiral environment), the chirality generation may occur in different parts of the substrate. For instance, when the enzyme is transforming a given functional group, a kinetic resolution of a racemic center may also proceed in the same molecule [18]. This elegant approach has been established for aldoxime dehydratases: while the aldoxime group is transformed to a nitrile, an adjacent racemate is simultaneously being resolved. A broad range of nitriles (comprising aromatic and aliphatic ones) have been successfully produced, leading to excellent conversions and enantioselectivities [19,20]. For instance, a range of α-substituted racemic aldoximes were converted in chiral nitriles using whole cells (overexpressing aldoxime dehydratases) and in aqueous media at low temperatures (<10 ℃) with enantiomeric excesses of up to >99%. Interestingly, aldoxime dehydratases displayed different enantioselectivity when *E* or *Z* aldoximes were used as substrates, providing options to synthetize both enantiomers with the same enzyme [19,20].

**Scheme 2:**
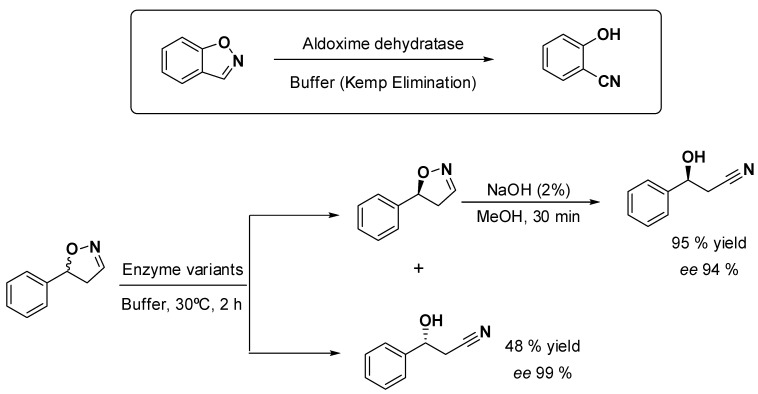
Aldoxime dehydratase-catalyzed Kemp elimination (above) and use of it to synthetize chiral β-hydroxy-nitriles, reported by the Asano group [21,22].

The Asano group has reported the Kemp elimination catalyzed by aldoxime dehydratases, and this finding was subsequently used to develop an elegant concept for the enantioselective ring-opening of racemic dihydroisoxazoles to afford β-hydroxy-nitriles. The obtained mixture of a chiral β-hydroxy-nitrile and the remnant optically active dihydroisoxazol could be straightforwardly separated (e.g., chromatography), and a further base-mediated opening of the untouched substrate afforded the two enantiomers of important building blocks for fine chemicals and pharmaceuticals. Reactions were performed in buffer at 30 ℃, and several enzyme variants were used (Scheme 2) [21,22]. Substrate concentrations were in the range of 100 mM (ca. 16 g L^−1^), which were converted in a 2 h reaction time. Albeit no further optimization has been reported yet, the developed proof of concept holds promising substrate-loading figures and conversions that probably can be improved in terms of productivity and reduced resource consumption [9]. It must be noted that, since the beginning of investigating these enzymes, the Asano group already worked with relatively high substrate loadings (in the range of 100 mM), giving hints on the synthetic potential of the aldoxime dehydratase platform [11,12,13]. Working at high substrate loadings is a must to align industrial interests with processes consuming less resources in terms of water and solvents [9].

**Scheme 3:**
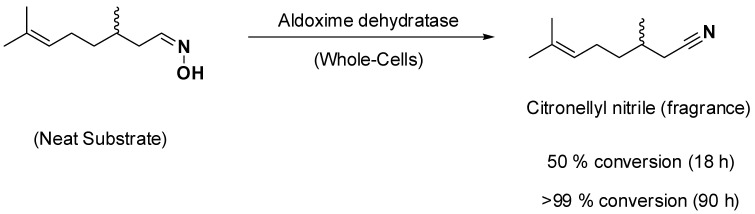
Synthesis of fragrances, such as citronellyl nitrile, using whole cells overexpressing aldoxime dehydratases and under solvent-free conditions [23].

Another important application of aldoxime dehydratases lies in the field of fragrances, as many terpene molecules bearing nitrile groups are utilized in these segments (e.g., nitriles derived from citronellol, geraniol, nerol, etc.), as a recent granted patent of BASF company discloses [23]. Starting from terpene aldehydes, the aldoxime can be straightforwardly formed by reacting with hydroxylamine. The reaction was conducted using whole cells overexpressing aldoxime dehydratases and in solvent-free conditions. That is, only citronellyl-oxime was added (neat) together with the whole cells, leading to full conversion (Scheme 3). Albeit reaction times are somewhat long (90 h for full conversion), and further process optimization may be necessary, working under solvent-free conditions enables the highest product formation possible as well as largely diminished waste generation associated with water or solvent used [9]. Moreover, avoiding solvents may also help circumvent regulations related to some markets and solvent use (e.g., food industry).

## 3. Toward the Production of Bulk Chemicals and Biorefinery-Like Approaches Using Aldoxime Dehydratases

As depicted above (Figure 1), nitriles are widely used not only for fine chemicals or pharmaceutical applications, but also for large-volume, low-value compounds like solvents, monomers (for polymerizations), commodities, etc. [1,2,3]. Given the promising substrate loadings that applications of aldoxime dehydratases have shown since the first characterization studies [11,12,13], the use of aldoxime dehydratases in bulk applications might offer industrial potential, provided that high productivities, together with efficient enzyme reuse, are aligned. In that respect, over the last years the Gröger group has established a platform with relevant applications of aldoxime dehydratases for non-chiral commodity-like syntheses.

With a focus on high substate loadings, the biocatalytic synthesis of aliphatic nitriles has been thoroughly studied as these compounds can be used as solvents as well as substrates for further reactions [24,25,26]. One promising approach is using whole cells overexpressing aldoxime dehydratases and performing the reactions in buffer (aqueous) media, with or without a co-solvent, to (co)dissolve the substrate. After pertinent optimizations, a range of aliphatic nitriles were synthetized with high yields and in short reaction times. As an outstanding example, *n*-octanaloxime was used as prototypical substrate for aldoxime dehydratases, and with stepwise loadings of up to 1.4 Kg L^−1^ high conversions to n-octanenitrile (>90%) were achieved in a 24 h reaction time. As reaction media, buffer with ethanol as a cosolvent (10% *v*/*v*) was used [24]. This reaction represents an outstanding example of a highly loaded biocatalytic reaction that may become useful for large-volume, low-value applications. Subsequently, the same group focused on the nitrile synthesis in non-aqueous media to provide versatile and compatible conditions for bulk chemicals. While first experiments using whole cells—either wet cells or lyophilized—were unsuccessful, the key to success was the immobilization of the whole cells in an “immobilized aqueous phase”, namely a polyacrylic acid derivative prone to absorb water. By means of these “superabsorbers”, aldoxime dehydratases (within whole cells) became stable and active, leading to the biocatalytic synthesis of aliphatic nitriles in organic media in continuous fashion with productivities of ~13 g nitrile h^−1^ L^−1^ [25,26].

Such performance of aldoxime dehydratases has stimulated the connection of these biocatalysts to petroleum and biorefineries, assessing how an enzymatic system can be integrated in a whole value production chain. In biorefineries, for instance, water is often playing a pivotal role, as biomass contains water and many reactions are performed in that media [4]. Conversely, in petroleum refineries, non-aqueous systems are the common path, and thus chemocatalysts have been designed to *evolve* to that environment. The demonstrated versatility of aldoxime dehydratases to work in both worlds offers high potentiality for future (bio)refineries. In that respect, multi-step processes starting from alkenes (e.g., 1-octene) and comprising metal-catalyzed hydroformylation (Rh-TPPTS catalyst), oxime formation, and biocatalysis for nitrile synthesis have been reported. To combine a multi-step system, careful optimizations were studied to avoid the presence of chemicals that may deactivate the subsequent catalysts. In that example, the first (hydroformylation) reaction was performed mostly in 1-octene (with 10% water, *v*/*v*) as reaction media. The subsequent reactions (oxime synthesis and the biocatalytic step) were then conducted in aqueous conditions [27]. This proof-of-concept illustrates the possibilities and the limitations of performing multi-step processes in one pot reactions. Aspects related to how to address the temperature difference of the reactions (heating/cooling) and downstream together with the heat integration remain as exciting challenges to be considered for a closed-loop practical application. Analogous cascade processes have been reported by combining (biogenic) fatty acids with cross-metathesis, hydroformylation, and use of aldoxime dehydratases to afford difunctionalized nitrile fatty acids that can find applications in polymer chemistry [28].

**Scheme 4:**
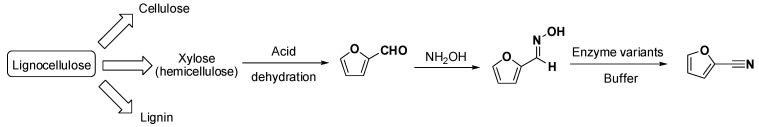
Conceptual approach for the production of 2-furonitrile starting from lignocellulose and incorporating an enzymatic step for the nitrile production [29,30].

Following the latter path and envisaging sustainable approaches, the area has been developed further by working with biogenic resources to produce nitriles. For instance, the synthesis of 2-furonitrile, starting from furfural, has been considered [29,30]. Furfural can be derived from the acidic dehydration of xylose (pentoses from hemicellulose), and it is a highly relevant platform chemical delivered from biorefineries [4,31]. Furfural can be condensed with hydroxylamine to yield the aldoxime that is subsequently converted by aldoxime dehydratases to 2-furonitrile, which has been used as sweetener as well as a building block for fine chemicals and pharmaceuticals. In this particular case, several aldoxime dehydratases variants were reported for the reaction (Scheme 4).

Albeit substrate loadings were not high in this case (10 mM), the example is relevant as it demonstrates that it is possible to evolve aldoxime dehydratases to particular substrates that may be linked to biorefineries. The above-described versatility of aldoxime dehydratases able to perform reactions both in aqueous and non-aqueous media may be particularly useful for complex biorefineries where crude effluents containing impurities, water, organic solvents, etc., are commonly found and must be used without downstream or purification to save energy and costs. In that respect, developing (bio)catalysts that can selectively generate value out of those otherwise challenging effluents appears of high importance for future processing chemical plants [4,31].

**Scheme 5:**
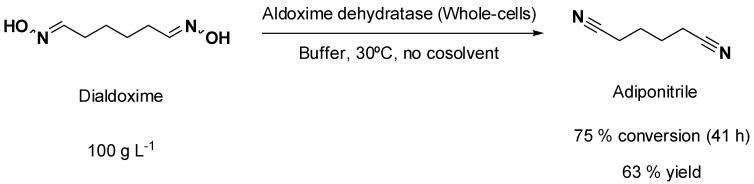
From α,ω-dialdoximes to adiponitrile using aldoxime dehydratases as biocatalysts [32].

In the same area, the Gröger group has studied the use of α,ω-dialdoximes to afford dinitriles with aldoxime dehydratases, taking the synthesis of adiponitrile as prototypical production [32]. This approach may have profound implications in biorefineries as adiponitrile is currently produced worldwide at a ton scale to be used in the polymer industry [4]. For this purpose, buffer was used with substrate loadings of up to 100 g L^−1^ and using whole cells overexpressing aldoxime dehydratases at 30 ℃. In initial procedures, DMSO was used as cosolvent, but it was observed that no cosolvent was needed to reach high conversion and yields (Scheme 5) [32]. This may provide improved environmental metrics due to the wastewater associated with the downstream when DMSO is removed [8,9]. In a more recent work, the concept has been further extended by starting with alcohols as biogenic raw materials and performing the ((2,2,6,6-Tetramethylpiperidin-1-yl)-oxyl) TEMPO-mediated oxidation, oxime formation, and biocatalytic step involving aldoxime dehydratases [33]. As in previous cases, a careful optimization of the multi-step approach was needed to secure that the different catalysts are not deactivated and that compatible conditions are found.

## 4. Concluding Remarks

The discovery and characterization of aldoxime dehydratases has opened many possibilities in organic synthesis. Nitrile synthesis is an important industrial reaction with a vast range of applications in areas like fine and bulk chemicals, pharmaceuticals, polymers, solvents, etc. Notably, aldoxime dehydratases have demonstrated to be versatile, accepting a broad substrate spectrum, and with rather high loadings (in some outstanding examples, more than 1 Kg substrate L^−1^ have been converted). Moreover, aldoxime dehydratases can perform reactions in different media, such as buffer, biphasic systems, solvent-free conditions (neat substrates), or in organic (co)solvents. This paper has discussed some relevant emerging cases of aldoxime dehydratases with particular emphasis on the applicability, industrial orientation, and environmental aspects related to them. Some in-depth comprehensive revisions of the topic can be found in the open literature [14,15,16,17]. In general, it appears that using whole cells overexpressing aldoxime dehydratases confers the enzymes with an extra protection that enables the efficient use of them in challenging non-aqueous media. Further work in this area is currently ongoing, such as the development of immobilization via super-absorbers and flow chemistry [25,26], or the recent incorporation of Pickering emulsions applied to aldoxime dehydratases [34]. Likewise, some variants have been already described in some cases, demonstrating the possibility of tailoring those enzymes to specific applications. However, the genetic design and quest for adequate aldoxime dehydratases variants seem to be still underrepresented and will surely provide novel applications in the future adapted to the desired molecules and systems. Finally, it must be noted that the possibility of linking biorefineries (through biogenic resources) with biocatalysis through aldoxime dehydratases will definitely contribute to pave the way to a more sustainable chemical industry in the future.

## References

[1] Hauddinger P., Glathaar R., Rhode W., Kick H., Benkmann C., Weber J., Wunschel H.-J., Stenke V., Leicht H. (2012). Ullmann’s Encyclopedia of Industrial Chemistry.

[2] Weissermel K., Arpe H.-J. (2004). Industrial Organic Chemistry.

[3] Pollak P., Romeder G., Hagedorn F., Gelbke H.-P. (2000). Nitriles. Ulmann’s Encyclopedia of Industrial Chemistry.

[4] Domínguez de María P. (2016). Industrial Biorenewables, a Practical Viewpoint.

[5] De Gonzalo G., Domínguez de María P. (2017). Biocatalysis: An Industrial Perspective.

[6] Sheldon R.A. (2016). The E factor 25 years on: The rise of green chemistry and sustainability. Green Chem..

[7] Sheldon R.A. (2017). Metrics of Green Chemistry and Sustainability: Past, Present, and Future. ACS Sustain. Chem. Eng..

[8] Van Schie M., Spöring J.D., Bocola M., Domínguez de María P., Rother D. (2021). Applied biocatalysis beyond just buffers—From aqueous to unconventional media: Options and guidelines. Green Chem..

[9] Domínguez de María P. (2021). Biocatalysis, sustainability and industrial applications: Show me the metrics. Curr. Opin. Green Sustain. Chem..

[10] Guajardo N., Domínguez de María P. (2019). Continuous Biocatalysis in environmentally-friendly media: A triple synergy for future sustainable processes. ChemCatChem.

[11] Xie S.-X., Kato Y., Asano Y. (2001). High Yield Synthesis of Nitriles by a New Enzyme, Phenylacetaldoxime Dehydratase, from Bacillus sp. Strain OxB-1. Biosci. Biotechnol. Biochem..

[12] Kato Y., Ooi R., Asano Y. (1999). A new enzymatic method of nitrile synthesis by *Rhodococcus* sp. strain YH3-3. J. Mol. Catal. B Enzym..

[13] Asano Y., Kato Y. (1998). Z-phenylacetaldoxime degradation by a novel aldoxime dehydratase from *Bacillus* sp. strain OxB-1. FEMS Microbiol. Lett..

[14] Chen K., Wang Z., Ding K., Chen Y., Asano Y. (2021). Recent progress on discovery and research of aldoxime dehydratases. Green Synth. Catal..

[15] Hinzmann A., Betke T., Asano Y., Gröger H. (2021). Synthetic Processes toward Nitriles without the Use of Cyanide: A Biocatalytic Concept Based on Dehydration of Aldoximes in Water. Chem. Eur. J..

[16] Gröger H., Asano Y. (2020). Cyanide-Free Enantioselective Catalytic Strategies for the Synthesis of Chiral Nitriles. J. Org. Chem..

[17] Betke T., Higuchi J., Rommelmann P., Oike K., Nomura T., Kato Y., Asano Y., Gröger H. (2018). Cover Feature: Biocatalytic Synthesis of Nitriles through Dehydration of Aldoximes: The Substrate Scope of Aldoxime Dehydratases (ChemBioChem 8/2018). ChemBioChem.

[18] Müller C.R., Pérez-Sánchez M., Domínguez de María P. (2013). Benzaldehyde lyase-catalyzed diastereoselective C-C bond formation by simultaneous carboligation and kinetic resolution. Org. Biomol. Chem..

[19] Metzner R., Okazaki S., Asano Y., Gröger H. (2014). Cyanide-free enantioselective synthesis of nitriles: Synthetic proof of a biocatalytic concept and mechanistic insights. ChemCatChem.

[20] Betke T., Rommelmann P., Oike K., Asano Y., Gröger H. (2017). Cyanide-Free and Broadly Applicable Enantioselective Synthetic Platform for Chiral Nitriles through a Biocatalytic Approach. Angew. Chem. Int. Ed..

[21] Miao Y., Metzner R., Asano Y. (2017). Kemp Elimination Catalyzed by Naturally Occurring Aldoxime Dehydratases. ChemBioChem.

[22] Zheng D., Asano Y. (2020). Biocatalytic asymmetric ring-opening of dihydroisoxazoles: A cyanide-free route to complementary enantiomers of β-hydroxy nitriles from olefins. Green Chem..

[23] Piatesi A., Siegel W., Baldenius K.U. (2015). Method for Biocatalytic Production of Nitriles from Oximes and Oxime Dehydratases Usable Therein. U.S. Patent.

[24] Hinzmann A., Glinski S., Worm M., Gröger H. (2019). Enzymatic Synthesis of Aliphatic Nitriles at a Substrate Loading of up to 1.4 kg/L: A Biocatalytic Record Achieved with a Heme Protein. J. Org. Chem..

[25] Hinzmann A., Adebar N., Betke T., Leppin M., Gröger H. (2019). Biotransformations in Pure Organic Medium: Organic Solvent-Labile Enzymes in the Batch and Flow Synthesis of Nitriles. Eur. J. Org. Chem..

[26] Adebar N., Gröger H. (2020). Heterogeneous catalysts “on the move”: Flow chemistry with fluid immobilized (bio)catalysts. Eur. J. Org. Chem..

[27] Plass C., Hinzmann A., Terhorst M., Brauer W., Oike K., Yazuver H., Asano Y., Vorholt A.J., Betke T., Gröger H. (2019). Approaching bulk chemical nitriles from alkenes: A hydrogen cyanide-free approach through a combination of hydroformylation and biocatalysis. ACS Catal..

[28] Hinzmann A., Druhmann S.S., Gröger H. (2020). Synthesis of bifunctional molecules for the production of polymers based on un-saturated fatty acids as bioderived raw materials. Sustain. Chem..

[29] Choi J.E., Shinoda S., Asano Y., Gröger H. (2020). Aldoxime dehydratase mutants as improved biocatalysts for a sustainable synthesis of biorenewable-based 2-furonitrile. Catalysts.

[30] Choi J.E., Shinoda S., Inoue R., Zheng D., Gröger H., Asano Y. (2019). Cyanide-free synthesis of an aromatic nitrile from a biorenewable-based aldoxime: Development and application of a recombinant aldoxime dehydratase as a biocatalyst. Biocatal. Biotransform..

[31] Stein T.V., Grande P.M., Leitner W., Domínguez de María P. (2011). Iron-Catalyzed Furfural Production in Biobased Biphasic Systems: From Pure Sugars to Direct Use of Crude Xylose Effluents as Feedstock. ChemSusChem.

[32] Betke T., Maier M., Gruber-Wölfler H., Gröger H. (2018). Biocatalytic production of adiponitrile and related aliphatic linear α,ω-dinitriles. Nat. Commun..

[33] Hinzmann A., Stricker M., Gröger H. (2020). Chemoenzymatic cascades toward aliphatic nitriles starting from biorenewable feedstocks. ACS Sustain. Chem. Eng..

[34] Rodríguez A.M.B., Schober L., Hinzmann A., Gröger H., Binks B.P. (2021). Effect of particle wettability and particle concentration on the enzymatic dehydration of n-octanaloxime in Pickering emulsions. Angew. Chem. Int. Ed..

